# Preventive or Potential Therapeutic Value of Nutraceuticals against Ionizing Radiation-Induced Oxidative Stress in Exposed Subjects and Frequent Fliers

**DOI:** 10.3390/ijms140817168

**Published:** 2013-08-20

**Authors:** Maria Teresa Giardi, Eleftherios Touloupakis, Delfina Bertolotto, Gabriele Mascetti

**Affiliations:** 1 IC-CNR Area della ricerca di Roma, Monterotondo scalo 00015, Italy; 2 Biosensor, Via Olmetti 44 Formello, Rome 00060, Italy; E-Mail: toulou_e@chemistry.uoc.gr; 3 Department of Chemistry, University of Crete, P.O. Box 2208, Voutes-Heraklion 71003, Greece; 4 Agenzia Spaziale Italiana (ASI), Viale Liegi 26, Rome 00198, Italy; E-Mails: delfina.bertolotto@asi.it (D.B.); gabriele.mascetti@asi.it (G.M.)

**Keywords:** ionizing radiation, low doses, oxidation stress, ageing, cardiovascular disease, cancer, retina degeneration, antioxidants, radio-protection

## Abstract

Humans are constantly exposed to ionizing radiation deriving from outer space sources or activities related to medical care. Absorption of ionizing radiation doses over a prolonged period of time can result in oxidative damage and cellular dysfunction inducing several diseases, especially in ageing subjects. In this report, we analyze the effects of ionizing radiation, particularly at low doses, in relation to a variety of human pathologies, including cancer, and cardiovascular and retinal diseases. We discuss scientific data in support of protection strategies by safe antioxidant formulations that can provide preventive or potential therapeutic value in response to long-term diseases that may develop following exposure.

## 1. Introduction

Exposure to ionizing radiation negatively impacts human health, increasing the risk of incidence of serious pathologies such as cancers and cardiovascular diseases [1–6]. Humans are often exposed to ionizing radiation from various terrestrial sources. In 1934, the International Commission on Radiological Protection (www.icrp.org) set the maximum safe radiation dose of 1–2 mSv/day, nevertheless, in the last ten years, an increase (by several mSievert) of the average absorbed dose per person has been registered in the United States, mainly due to the extensive use of detection imaging techniques [2]. Frequent fliers and Air Force personnel are also exposed to low doses of ionizing radiation. Frequent fliers may even receive the same dose as radiation personnel [7]. The Earth’s magnetic field and atmosphere effectively shield humans against exposure to ionizing cosmic radiation. However, doses received during airplane or space flights are between ten and one hundred times higher, respectively, than on Earth.

Human space exploration missions to the Moon and Mars are being planned. These long-term missions will lead humans to live outside the protection provided by the atmosphere and the geomagnetic field of the Earth. It is well known that deep space is permeated by complex natural radiation. At high energy levels in the millions of electron volts, radiation particles have enough energy to ionize the atoms in materials through which they propagate. At lower energy levels, in the thousands of electron volts, their effects range from charge accumulation on surfaces to material degradation. Natural radiation consists of electrons and protons trapped by planetary magnetic fields, protons, and a very small fraction of heavy nuclei. The primary cosmic beam, consisting of approximately 85% protons and 15% heavy nuclei, is partially converted into secondary neutrons by collisions with matter. In turn, these secondary neutrons can produce additional radiation types of various energy levels. In space, the flux of radiation varies unpredictably between low and high doses generated by sun storms and supernova explosions outside our galaxy [8–10].

The possibility of developing degenerative diseases as a consequence of radiation exposure during deep space exploration should be considered before attempting exploratory missions. Furthermore, it should be also taken into consideration that radiation exposure could also determine late effects, extending the risk for astronauts with ageing, several years after their return to Earth. It has been reported that a mission to the Martian surface may require 500 days in deep space and for a middle-aged man, estimates of the corresponding risk of death from oxidative damage due to space missions vary widely, between 1% and 13% [11,12].

Radiobiologists have debated the potential risks of oxidative stress induced by low radiation doses in humans for several years. Some suggested that the health risks of doses of less than 10 cGy cannot be measured, while others claimed that no dose of ionizing radiation can be considered completely safe and that the use of radiation must always be determined on the basis of risk versus benefit [12–15]. However, the former idea ignores the effect of interaction between radiation-induced stress and other stress conditions that may enhance diseases and the risk of oxidative stress-induced cancer, particularly in ageing patients [5,6,16–18].

Estimation of chronic low-dose radiation effects on health is still incomplete and challenging. In recent years, several committees have been appointed to assess the damaging effects on health determined by exposure to low levels of ionizing radiation. In 2006, the National Academy of Sciences in Washington emphasized the importance of including highly-exposed people in the research to reveal the effects of long-term low-level radiation exposure in ageing population [13]. In 2008, the United Nations Scientific Committee on the Effects of Atomic Radiation recommended paying more attention to the study of cancer and other non cancer diseases induced by low radiation levels, as well as to circulatory diseases, calling for epidemiological studies on the subject [14]. In 2011, the International Commission on Radiological Protection stated that particular attention should be paid to the effects of radiation on eye cataracts and cardiovascular disease, as effects on these organs occur at lower doses compared to those previously reported [15].

Chromosomal aberrations due to radiation were observed by Müller in the 1930s, demonstrating that high-energy radiation causes large translocations with very rare point mutations [19]. In addition, ionizing radiation-induced oxidative stress can also lead to multiple single point mutations or small nucleotide deletions, acting indirectly on DNA and producing chronic effects.

Both mechanisms generate cancer and long-term diseases, and according to specific literature, overlapping effects can be evidenced depending on the energy and time of exposure to a radiation source [3,20,21]. It is widely accepted that the risk of exposure to ionizing radiation at low doses and dose rates is dominated by cancer and that the related mechanisms are driven by mutational damage to DNA. However, a role for non-DNA-targeted effects has been postulated [20–22].

Atherosclerosis is the main cause of coronary heart disease and strokes, the two major causes of death in developed countries. Though initiation of atherosclerosis was attributed mainly to lipid accumulation within the arterial walls, it is now widely accepted that inflammation plays a vital role in the initiation and progression of the disease [23–27]. High radiation doses to the heart, coronary, carotid and other large arteries received during specific radiation therapy procedures induce tissue damage, resulting in increased risk of circulatory diseases; the underlying biological mechanism is the high level of cell killing, leading to pro-inflammatory effects and micro-vascular damage [26,27]. The essential question is whether low doses of ionizing radiation can increase the risk of this disease, as indicated by some epidemiological studies which also highlighted different action mechanisms compared to those active in high-dose exposure. There is emerging evidence of excess risk of cardiovascular disease at much lower radiation doses and occurring a long time after radiation exposure in Japanese atomic bomb survivors and in various occupationally-exposed groups [27–31].

It is important to note that Japanese atomic bomb survivors have been found to display increased levels of the pro-inflammatory cytokines IL-6, CRP, TNF-α and INF-γ, but also increased levels of the anti-inflammatory cytokine IL-10. A rise in erythrocyte sedimentation rate, in IgG, IgA and total accumulation levels of immunoglobulin has been observed [27,32], which indicates chronic inflammation.

Recent studies have also examined the risk of cardiovascular disease occurrence in humans exposed to various sources of radiation, such as dirty bombs used by terrorists, medical treatments, radio-surgery, medical imaging and space flights. The evidence suggests a strong association between cardiovascular disease, ageing and exposure to low-to-moderate levels of radiation [5,6]. Other clinically important consequences of exposure to radiation are retina degeneration and eye cataracts, which were previously thought to be deterministic or tissue reactions but are currently recognized as possibly stochastic in nature and determined by much lower radiation levels than previously believed [33–35].

The hypothesis that low radiation doses induce oxidative stress has not yet been demonstrated at a mechanistic level. However, low radiation doses have been found to be significant in long-term studies of the after-effects of the Chernobyl accident [36–38]. At low dose rates recorded in the Chernobyl area, in the order of 400 nGyh^−1^, radiation effects on sperm motility in birds have been reported. Some of the studies on birds showed that differences in habitat structure are also responsible for altered concentration of antioxidants, together with the direct effects of oxidative stress induced by radiation [39–41].

It has recently been reported that the Fukushima accident has caused physiological and genetic damage to *Zizeeria maha* butterflies. Low-dose exposure in the 3–55 mSv range, experimentally reproduced *in vitro*, showed similar effects. The data obtained from this research revealed that these modifications were due to random genetic mutations caused by the artificial radionuclides generated by the Fukushima disaster [42]. Space flight experiments on mice demonstrated that several genes, involved in scavenging reactive oxygen species and anti-inflammation function, were up-regulated after the mission. However, these studies made no distinction between stresses correlated to ionizing radiation or to absence of gravity [43]. It has been demonstrated that neutron irradiation in Arabidopsis plants affects the expression of several senescence-related genes inducing the up-regulation of the SAG12 and SAG13 genes, involved in senescence, and of CAT1, CAT3 and FeSOD1, involved in the reduction of free radicals [44].

It has been reported that up to 70% of cancer survivors also experience various health problems and a wide array of diseases, even many years after radiation exposure. It seems that the accumulation of dysfunctional senescent cells in tissues, combined with a reduction in the proliferative potential of progenitor/stem cells (as a result of their apoptosis/senescence), may account for the development of long-term diseases [5,45].

Multiple pathways are involved in the initiation and advancement of degenerative diseases due to irradiation. We will consider the roles of reactive oxygen species in detail.

## 2. ROS and Antioxidant-Oxidant Levels

Absorption of ionizing radiation by living cells can produce chemical and biological modifications directly. It can also act indirectly through radiolysis of water, thereby generating reactive chemical species that may damage DNA, proteins and lipids [46]. The early biochemical changes occurring during or just after radiation exposure were thought to be a consequence of ionizing radiation effects. However, it has been also demonstrated that oxidative stress causes changes for several months after exposure, possibly due to further generation of reactive oxygen species and nitrogen radicals (ROS and RNS, respectively). Interestingly enough, these modifications occur both in the exposed biological cells and in their progeny. Oxidative stress may also spread from targeted cells to non-targeted bystander cells by means of intracellular control mechanisms [47]. It is important to note that the progeny of bystander cells undergo modifications in their biochemical patterns and metabolism, presenting oxidative damage, including protein, lipid peroxidation, carbonylation, mutations and neoplastic transformations [48]. The propagation of oxidative stress in progeny cells has significant clinical consequences for long-term diseases with ageing patients, such as induction of a second tumor after radiotherapy [45,49].

The relative yield of ROS and RNS is strongly dependent on the different types of radiation. The extent and nature of induced DNA damage also depend on the yield of these products and their local concentrations [16,49,50]. ROS and RNS can attack cells, causing several modifications, including DNA stream breaks, base damage, telomere dysfunction, protein and lipid cross-linking, as well as destruction of sugars [16,50,51]. Although the spectrum of generated ROS is similar to that produced by metabolic processes, differences may exist in the micro-distribution of ROS generated during irradiation. Thus ROS produced under physiological conditions are important signaling molecules which regulate biochemical cellular processes, while excess ROS, produced by ionizing radiation, are toxic [49,50]. NAD(P)H-oxidases, lipoxygenases, nitric oxide synthases, xanthine oxidase, microsomal cytochrome P-450, and mitochondrial electron transport chains are the main sources of ROS [16,50–53].

Recent evidence demonstrates that modulation of oxidative metabolism may persist after the decay of primary and secondary chemical species, disrupting the stability and activity of DNA repair processes. As a result, *de novo* DNA damage continues to occur, thus influencing the regulation of gene expression. Therefore, understanding the process that leads to perturbations in oxidative metabolism would contribute to the development of strategies to counteract oxidative stress (e.g., by administration of antioxidants) in order to control the progression of cancer and other degenerative diseases.

The relationship between concentration of antioxidants and oxidative stress has been extensively studied: for example, studies in higher plants have shown higher content of antioxidant enzymes with exposure to low rates of radiation. Other reports have demonstrated no changes in antioxidant enzymes in higher plants or animals, although a number of studies have observed a modification in the content of metabolites induced by reactive oxygen [36,51–55].

Interestingly, mathematical models have been developed to measure the amount of radiation capable of inducing oxidative stress at the dose rates of the contamination densities found in Chernobyl. The conclusion of the mathematical model was that low doses of ionizing radiation produce only a small quantity of ROS unable to affect antioxidant concentrations in cells [54]. However, in the last 15 years, evidence has suggested that a small local ROS modification may cause altered signal transductions [55,56].

Since critical steps in signal transduction mechanisms are redox-regulated, the efficacy of different antioxidants on the molecular mechanisms implicated in human diseases and in ageing should be the object of further studies, aiming to explore the potential of antioxidant therapy [55].

Although these issues have been discussed in several studies, there is still a need to develop scientifically rational approaches to provide countermeasures against ionizing radiation stress. This work summarizes scientific data in support of new antioxidant formulations and nutraceuticals that could provide biological protection in humans.

## 3. Protection Strategy in Humans and the Search for Biological Radiation Protection

Several studies aiming to protect normal tissue were performed when X-ray therapy was being developed. Initial attempts at radiation protection involved physical countermeasures such as shielding unexposed areas with lead, reducing exposure time, and increasing the distance between the radiation source and patients/workers. Although these procedures are all beneficial, they have several limitations. Clearly, none of them is feasible during space flights, since space radiation is highly penetrating and it is not technically possible to adopt convenient shields.

In order to address the problem of radiation-induced oxidative stress protection, international agencies have introduced the concept of limiting doses to the lowest possible for humans [4]. Nevertheless, the introduction of novel strategies may reduce the risk of radiation damage in humans, particularly in subjects exposed during flights.

Extensive radio-biological research has discovered various chemical compounds which, when used before exposure to radiation, may protect against oxidative stress [56–78]; for example we may list SH-compounds such as cysteamine, cystamine and aminoethylisothiourea dihydrobromide, and amifostine, a cysteamine analogue [57–60]. The use of these protective compounds has been suggested by the observation that mitotic cells, which are highly sensitive to radiation, have a low level of SH-compounds, whereas S-phase cells, which are highly resistant to radiation, have a high level of SH-compounds. The relevance of SH-compounds in protection has also been demonstrated by the fact that increasing their intracellular levels in mitotic cells leads them to become radioresistant as S-phase cells [57–59].

It has been demonstrated that vitamin E and selenium, as well as the combination of these two compounds, reduce transformations induced by radiation in cell cultures [60–64]. Both vitamins C and E have been shown to decrease chromosomal damage, mutations and apoptosis in mammalian cells, while β-carotene protects against neoplastic transformation induced by radiation *in vitro* [63]. Other important studies have shown that vitamin A and *N*-acetylcysteine may be effective against carcinogenesis caused by radiation [60].

Several studies on animals also support the use of antioxidants for effective radiation protection in humans [64–73]. Alpha-lipoic acid considerably increases the lethal dosage of radiation in mice, while vitamins A, B and C and also β-carotene have been known to protect rodents and various types of animals against high radiation levels [64–73].

l-seleno methionine and various types of antioxidants, such as vitamins C and E, glutathione, α-lipoic acid, *N*-acetylcysteine, and Q_10_ co-enzyme can protect mice and humans against oxidative stress [74–78]. A combination of vitamin E and α-lipoic acid was more effective than the individual agents in decreasing oxidative damage in children of the contaminated Chernobyl area [76–78]. β-carotene has been shown to protect against mucositis induced by head and neck cancer radiotherapy. A synergistic effect of dietary antioxidants capable of protecting normal tissue during radiation therapy to a greater extent than the individual components has been observed [79–81]. SH-compounds have been found to be toxic in humans, while compounds increasing glutathione, such as α-lipoic acid and *N*-acetylcysteine, which are less toxic to humans, are now commonly used to protect against radiation damage [60,77,81–85].

A number of vitamin formulations for personnel exposed to radiation are marketed by Premier Micronutrient Corporation, Nashville, TN. However, several efforts are underway to discover the perfect radioprotector(s) with the ability to reduce or delay the consequences of genomic instability induced by radiation [83–86].

Dietary antioxidants may protect against oxidative damage induced by radiation sources, but data from exposed human populations are limited. Interestingly, the efficacy of antioxidant strategies for biological radiation protection has recently been tested in frequent fliers [7]. The association between the frequency of chromosome translocations, as a biomarker of cumulative DNA damage, and intakes of antioxidants has been analyzed in about a hundred airline pilots. Intakes of vitamin C, β-carotene, β-cryptoxanthin and lutein-zeaxanthin were associated to modifications of translocation frequency [7].

## 4. Multiple Antioxidants against Stress Induced by Ionizing Radiation

Several studies and approaches support the therapeutic effect of antioxidant supply against radiation damage in humans as reported above. Based on data published concerning antioxidants, it may be possible to discuss the preventive or potential therapeutic value of antioxidants which could provide biological protection against long-term, low-dose radiation effects, also occurring at specific human organ level.

Plants and other photosynthetic microorganisms, mainly algae, are capable of synthesizing essential primary metabolites, such as carbohydrates, lipids and amino acids. The high quantity of omega-3 in fish is due to their consumption of photosynthetic algae that synthesize and accumulate high levels of unsaturated fatty acids [87,88]. In addition, plants produce a remarkable variety of low-molecular-weight organic compounds, termed secondary metabolites, usually with unique and complex structures. They are endowed with functional activity that provides benefits in humans beyond basic nutrition and are already very important industrial and economic sources of several nutraceutical formulations [87]. Such indications provide a rationale for subjecting plant antioxidant compounds to further scrutiny in order to identify the molecular basis of oxidant/antioxidant action against ionizing radiation.

Various reviews focused on the *in vivo* radioprotective efficacy of vitamins, minerals, caffeine, genistein and melatonin, and their influence at various endpoints of radiation damage [82–86,89–91]. Exhaustive reviews have already reported the use of vitamins and herbs as natural sources of protection against ionizing radiation [84,89]. Arora *et al*. have also gathered the most promising plants already widely used in traditional systems of medicine and that have produced significant radioprotection in both *in vitro* and *in vivo* model systems [92].

Several papers have recently reported the use of phytochemicals against oxidative stress. However, additional studies are needed to establish their efficacy in the presence of ionizing radiation. The most interesting groups of phytochemical compounds, currently marketed by the functional food industry, and that could have a positive effect on human organs, according to epidemiological studies, have also been discussed. Polyunsaturated fatty acids, phytosterols, phenolic compounds, xanthophylls, alliin, allicin, glucosinolates and capsacinoids are briefly described in this section, particularly in relation to their potential protective action against induced stress due to ionizing radiation and its long-term effects, such as induction of cardiovascular, cancer and retinal diseases. Table 1 reports the effects of antioxidants on gene expression and their healing effects on induced diseases.

### 4.1. Polyunsaturated Fatty Acids and Phytosterols

A strong correlation between cardiovascular disease, ageing and exposure to low-to-moderate levels of radiation has recently been presented by Baker *et al*. [6]. The neurological system is highly affected in space, while the risk of cardiovascular disease is present after astronauts return to Earth, with ageing [9,11,12].

Polyunsaturated fatty acids (PUFAs) and phytosterols have been shown to act at the cardiovascular level, ameliorating the metabolic patterns of endogenous antioxidants and lipids and membrane fluidity. It has been suggested that integration with PUFAs and phytosterols is important mainly to protect the neurological and cardiovascular systems of exposed subjects and frequent fliers. PUFAs are long-chain unsaturated carboxylic acids with more than one double bond. The preferred nomenclature when dealing with PUFAs in the field of nutraceuticals and functional metabolites is “*n-x*” or “ω*-x*” which originates from the typical characteristics of these compounds, where *n* or ω indicates the terminal methyl group of the PUFA chain while *x* indicates the position of the carbon-carbon double bond. Linolenic, arachidonic, docosahexaenoic acid (DHA) and eicosapentaenoic (EPA) acids are considered essential nutrients as they cannot be produced by the body and need to be supplied by a diet. Recent dietary data suggest an optimal ω-3/ω-6 ratio of 4 to 1 or lower. In actual fact, the ratio in modern diets is typically in excess of 10 [99–101]. Fish, algae and green vegetable oils are important sources of omega 6 and 3 [87,88,102].

Intake of the omega-3 fatty acids EPA and DHA by adults has been correlated with a decreased risk of cardiovascular disease [103–105], as they reduce blood pressure, improve the endothelial function and slow down the formation of atherosclerotic plaques. In addition, a high concentration of omega-3 leads to high membrane fluidity, with a consequent increase in serotonin transport. Omega-3 fatty acids also play a role in the activity of the central nervous system, increasing cognitive development and memory, as they are involved in neural development [105–108]. However, this effect on neural development is currently subject to debate [109].

Phytosterols exist as naturally occurring plant sterols that have a chemical structure similar to cholesterol except for the presence of an extra methyl or ethyl group. It is thought that phytosterols reduce cholesterol absorption, although the exact mechanism is not known. Phytosterols play an important role in regulating cardiovascular disease and present anti-cancer activity [110].

### 4.2. Organosulfur and Nitrogen Compounds

Alliin (cysteine sulfoxide) and allicin (cysteine thiosulfinate) are two important organosulfur compounds that can be isolated from *Allium sativum*, in which they are present in high concentrations [111]. Allicin is formed by the action of the enzyme alliinase on alliin, which is a derivative of the amino acid cysteine. This action induces the preservation of cardiac activity by means of a ROS-dependent mechanism, involving multiple intracellular signaling; such properties indicate a possible protection against cardiovascular disease in subjects exposed to ionizing radiation. Allicin protects against cardiac hypertrophy and fibrosis via attenuating reactive oxygen species-dependent signaling pathways, and ameliorates ageing-induced cognitive, learning and memory deficits by enhancing Nrf2 antioxidant signaling pathways [112–114]. Organic-sulfur compounds also inhibit tumor cell growth and cellular proliferation, influencing DNA repair mechanisms and reducing chromosomal aberrations. Diallyl trisulfide and diallyl sulfide stimulate T-cell proliferation and macrophage cytotoxicity on tumor cell lines, and increase the activity of detoxifying enzymes. Some reports have revealed that their inhibitory effect on cardiac hypertrophy is mediated by blocking the activation of ROS-dependent extracellular regulated kinases (ERK1/2), c-Jun *N*-terminal kinases (JNK1/2) and serine/threonine kinase (AKT). Other experiments have shown that allicin reduces inflammation and resulting fibrosis by inhibiting the activation of nuclear factor-κB. In addition, they are active against fungi, viruses and bacteria [113–115].

Capsaicinoids belong to the nitrogen-containing plant secondary metabolites, specifically to the family of aromatic fatty amides produced by chili peppers [87,116]. The main capsaicinoid in chili peppers is capsaicin, which typically accounts for approximately 70% of the capsacinoid content. The second most abundant species is dihydrocapsaicin, which forms about 20% of said content. Capsaicin and other capsaicinoids possess important pharmacological properties as they influence the peripheral part of the sensory nervous system, thereby reducing pain. However, their potential role in ionizing radiation protection is suggested by numerous studies focused on the anti-carcinogenic and anti-mutagenic properties of capsaicin [116].

Glucosinolates are nitrogen- and sulfur-containing secondary plant metabolites occurring at high concentrations in all cruciferous plants. Approximately 120 glucosinolates are known: all of their molecules share a common chemical structure consisting of a sulfonated moiety, a β-d-thioglucose group and a variable side chain derived from one amino acid. Glucosinolates can be classified into three chemical groups depending on their amino acid precursor. Aliphatic glucosinolates derive from alanine, leucine, isoleucine, methionine or valine; aromatic glucosinolates derive from phenylalanine or tyrosine; indole glucosinolates derive from tryptophan. Most of the biological activities of glucosinolates can be attributed to the action of their hydrolysis products; the isothiocyanate sulforaphane derivative of 4-methylsulfinylbutyl glucosinolate and other isothiocyanates may prevent tumor growth, blocking the progression of the cell cycle, and promoting apoptosis [87,117]. These phytochemicals may be useful as general protectors against radiation.

### 4.3. Polyphenolic Compounds

Polyphenols include several thousand compounds such as flavonols, flavones, catechins, flavanones, anthocyanidins and isoflavonoids [118–122]. Flavonoids derive from 2-phenyl-1,4-benzopyrone, commonly known as flavone. In plants, flavonoids are involved in various functions such as pigmentation, attracting and repelling insects as well as plant protection from herbivores. Their beneficial health effects in humans are thought to be due to their antioxidant, free-radical scavenging action and metal-chelating ability. Therefore, the ability of flavonoids to inhibit lipoprotein oxidation plays an important role in preventing cardiovascular diseases. Several *in vitro* and *in vivo* experiments have shown that flavonoids may inhibit carcinogenesis, by affecting the molecular events in the initiation, promotion and progression states of cancer. Moreover, some flavonoids are thought to play a role in inhibiting the production of inflammatory mediators such as prostaglandins, leukotrienes and nitric oxide [87,122].

Cell exposure to phenolic antioxidants induces the expression of genes encoding antioxidative and Phase II detoxification, which are regulated by a specific enhancer, the so-called antioxidant response element (ARE). The transcription factor Nrf2 plays the role of central protein that interacts with the ARE to activate gene transcription in response to oxidative stress. Some reports indicate that the sequence of the ARE, the structure of the inducers and the type of cells determine the response of the enhancer in a particular gene [123,124].

Curcumin (diferuloymethane) is an important example of polyphenols present in tropical plants. *Curcuma longa* (Linn.), a root related to ginger responsible for the yellow color of turmeric, is thought to possess anti-inflammatory and antioxidant properties. Curcumin has been used for hundreds of years as a medical component in traditional medicine [87]. The ability of curcumin to inhibit cyclooxygenase (COX-1 and COX-2) enzymes and to reduce the activation of NF-κB (see Table 1) [125] is of particular importance.

In addition to its ability to scavenge ROS, this yellow compound also reduces cell growth by inhibiting the activity of protein kinases. Moreover, curcumin contains two electrophilic α,β-unsaturated carbonyl groups, which can react with nucleophiles (e.g., glutathione). Based on its Michael reaction acceptor activity and its electrophilic characteristics, curcumin has recently been shown to induce Phase I and Phase II detoxification system activities. Other reports have shown that it plays a role in increasing the activity of γ-glutamyl-cysteinyl synthetase and of GSH-linked detoxifying enzymes. Furthermore, curcumin induces heme oxygenase-1 (HO-1) expression in vascular endothelial cells and in cultured hippocampal neurons, as well as in rat astrocytes [126,127]. The ability of curcumin to induce HO-1 has been suggested as an explanation for its strong antioxidant and anti-inflammatory properties, together with its radical scavenger effect [127,128].

These properties suggest that curcumin may have the ability to improve low-dose radiation-induced oxidative health damage, although no in-depth studies have been conducted to date. This suggestion is supported by the observation that mammary and pituitary tumors in rats induced by ionizing radiation are inhibited by curcumin supplied after radiation exposure [126,128].

Resveratrol has been also shown to have a radiation protective effect, significantly reducing the frequencies of chromosome aberrations in mice treated with 3 Gy of gamma-radiation [129].

## 5. Case Study: Xanthophylls against Ionizing Radiation and in Protection of Astronauts

Carotenoids are organic lipid-soluble pigments found in the chloroplasts and chromoplasts in plants and certain types of algae [87,88]. The most abundant type of carotene is β-carotene. One of the most important and well-known members of the carotenoid family is lycopene, an intermediate for the biosynthesis of many carotenoids. Xanthophylls include several compounds, such as zeaxanthin, neoxanthin, violaxanthin and α- and β-cryptoxanthin, oxidized derivatives of carotenes. Xanthophylls, such as astaxanthin, anteraxanthin, criptoxanthin, violaxanthin and rubixanthin, have an important role in protecting the visual power train. Both carotenes and xanthophylls belong to the category of polyisoprenoids and contain 40 carbon atoms formed by the condensation of eight isoprene units. The presence of conjugated double bonds gives carotenoids the ability to absorb light in the visible region of the spectrum. Different levels of hydrogenation and the introduction of oxygen-containing functional groups in the left- and right-end chains create a large family of over 600 natural compounds [87]. In plants, lutein and zeaxanthin accumulation levels are lower compared to carotenes. Lutein and zeaxanthin are the only carotenoids which are found in abundance in the human body, in the macular region and the crystalline lens of the eye. The human body is not capable of synthesizing xanthophylls and must obtain them through diet or supplementation [130–132]. The mechanisms related to their antioxidant action include targeting tumor cell growth, protection against external factors (for instance by filtering out excess light), targeting xenobiotic metabolism, and changing the antioxidant capacity of cells and the metabolic pattern of endogenous antioxidants, lipids and membrane fluidity [131,132]. An analysis of the effects of such compounds is given in Table 1.

A significant problem for prolonged space flights is exposure to ionizing radiation resulting in oxidative stress [133]. One of the organs most affected by cosmic radiation is the visual system, particularly the central and peripheral photoreceptors of the retina. Astronauts experience the phenomenon of perception of light flashes due to the impact of cosmic radiation [134,135]. The result is that their vision during night explorations is particularly disturbed. Recent studies conducted on the International Space Station suggested that a unique ionizing heavy particle may strike one or more photoreceptors in the retina, causing damage to other eye tissues, such as the lens. The mechanism of oxidation at retina level is not known in detail. One hypothesis is that damage is generated in the lens epithelial cells, including the destruction of normal cell life cycles, apoptosis, abnormal differentiation of cells and cellular disorganization [134–136].

As mentioned above, lutein and zeaxanthin are concentrated in both the macula and the lens of the human eye and are referred to as macular pigments (MPs). The effects of MPs on the human body include improvement of visual function and protection from photo-induced damage. Epidemiological studies have found a correlation between accumulation levels of lutein and zeaxanthin in eye tissues, serum and blood plasma, with reduced incidence of oxidative stress [137,138].

Two different mechanisms underlie retinal damage caused by radiation. The first is mediated by rhodopsin, which is a protein responsible for the binding of photoreceptors, which undergoes a bleaching process. The second is mediated by lipids, which undergo a process of photooxidation. Oxidative processes associated with ageing may also increase the concentration of lipofuscin and other non-degradable molecules which are not eliminated by exocytosis [139]. An excessive accumulation of lipofuscin may interfere with the function of epithelial retinal pigmented cells through the generation of free radicals. Lutein and zeaxanthin have polar groups at the ends of the molecule that stretch out to cell membrane lipids, interacting with the radicals outside the membrane. Lack of macular pigments with an antioxidant effect has been shown to accelerate the formation of lipofuscin in the cells [139,140]. Here, granules are formed which contain lipids that are generally brown-colored. Some experimental evidence demonstrates that lutein and zeaxanthin possess protective effects against oxidative damage induced by radiation. The function of these pigments is to improve eyesight by quenching free radicals and, in so doing, acting as antioxidants to protect the macula from oxidative damage. In addition, lutein and zeaxanthin protect eyes against photo-induced damage by shielding against ultra-violet light and potentially harmful shortwave radiation. Significant correlations have been found between lutein and zeaxanthin concentrations in ocular tissues, serum and plasma, and a possible reduction in the risk of macular degeneration [141,142].

Algae are rich in pharmacologically-active natural products and nutraceuticals. *Chlamydomonas reinhardtii* is a photosynthetic alga and is widely used as a model system for the study of photosynthetic processes. It has been used widely for the production of nutraceutical compounds under extreme conditions such as those experienced during space flight [143,144]. *Chlamydomonas reinhardtii* also possesses an orange organelle called eyespot, containing rhodopsins and macular pigments lying in a bed of thylakoids, involved in the perception of light. It is fascinating to observe that the human retina has maintained a similar structure to that of the algal eyespot [145] during its evolution. We analyzed the resistance to space stress of *Chlamydomonas reinhardtii* in flight, using genetic mutants which accumulate different quantities of the macular pigments zeaxanthin and lutein in their eyespots; analysis of the expression of the genes involved in the production of macular pigments and in the assembly of the algal eyespot indicates protection against space ionizing radiation by lutein and zeaxanthin is stronger than by carotenes [146]. This observation reflects the results of Yong *et al*., that have shown that supplementation of lutein/zeaxanthin in frequent fliers reduces chromosomal aberrations [7].

## 6. Conclusions and Perspectives

The information obtained so far from previous studies clearly indicates that critical steps in human biochemical pathways are sensitive to the actions of oxidants/antioxidants.

Dietary antioxidants have been consumed by people for decades and, within certain dose limits, no toxicity has been reported [87]. However, controversial effects of polyphenols have been demonstrated. Resveratrol, which is a stilbene derivative, for example, can improve lipid profile and glucose levels in the case of high-fat diets, but produces hepatic oxidative stress in standard-fed diets [147]. Epigallocatechin-3-gallate, which is the main polyphenolic constituent of green tea, is known for its antioxidant properties. However, when ingested in excess, it can promote the formation of radical species, causing oxidative cell damage [148]. In addition, the breakdown products of phenolic compounds can act as anti-nutritional factors in the diet [149].

In spite of the beneficial effects of capsaicin metabolites on stomach cancer, with regard to chili intake, one study confirmed that high consumption of chili pepper by rats leads to a greater risk of contracting gastric cancer [87,150]. A number of negative effects have been reported in relation to glucosinolates, mainly regarding their effect on the thyroid gland in various animals, as the ingestion of overdoses causes an abnormal absorption of iodine by the gland, causing hypertrophy and goitre [97].

Findings from prospective cohort studies suggest that an inverse association between carotenoids and lung cancer may exist. Poor nutritional habits (e.g., absence of fruit and vegetables from the diet), together with smoking, have been associated statistically with a significantly elevated overall risk of lung cancer [151]. Adverse effects have been also reported in diabetic patients subjected to excessive β-carotene food intake [152]. To our knowledge, no toxicity reactions have been reported for lutein and zeaxanthin assumption of more than 40 mg/day [153]. Consumption of phytosterols may lead to the reduction of blood levels of carotenoids that could be overcome by increasing the intake of carotenoid-rich foods [110,154].

Mechanisms related to phytochemical action include several aspects, such as protection against environmental factors by targeting xenobiotic metabolism, increasing the antioxidant capacity of cells, ameliorating the metabolic pattern of endogenous antioxidants, lipids and membrane fluidity and targeting tumor cell growth [93–96,98,155–161].

The ability of various antioxidants to trigger molecular mechanisms favorably towards a defense response, induced by long-term ionizing radiation exposure, should be a critical determinant for their use in clinical studies. Experiments with radiation sources and antioxidant formulations cannot be performed in humans for obvious ethical reasons. The proposed antioxidant ingredients have been shown to provide protection against oxidative stress in humans subjected to various forms of stress or injured accidentally by radiation and/or in laboratory experiments. Thus, the proposed recommendations may prompt future studies among radiation-exposed individuals designed to further demonstrate their efficacy in reducing health risks. Experimental studies showing that plant antioxidants induce apoptosis in cancer cells, but not in normal cells, are largely performed *in vitro*, and it is only possible to extrapolate/speculate upon an *in vivo* correlation. Therefore, further studies are required with regard to therapies for ionizing radiation.

Considering the data, we suggest that a cautious and judicious use of antioxidants that help radiation-exposed subjects to maintain a good quality of life may be helpful and we can only encourage people to refrain from ingesting high levels of antioxidant supplements.

The use of antioxidants is governed by EU legislation to control and safeguard the health of the population. In 2006, EC Regulation 1924/2006 required that the effects on health of food supplements and nutraceutical ingredients used/marketed by companies must be proven. In 2008, EC Regulations 109/2008 and 353/2008 further implemented Regulation 1924/2006 by requiring scientific evidence from human studies to be submitted to the European Food Safety Authority/European Medicines Agency (EFSA/EMEA), in order to obtain specific authorization for risk factor reduction claims. However, several indications provide a strong rationale for subjecting antioxidant compounds to further scrutiny in order to reveal the molecular basis of oxidant/antioxidant action and to protect humans against low radiation doses.

It is important to comply with the physical principle of protection against ionizing radiation and the principle of “as low as reasonably achievable” or ALARA [162]. Combining this concept with the preventive therapeutic value provided by multiple antioxidants, it might be possible to reduce health risks of ionizing radiation, in particular with low doses of radiation, irrespective of how small that risk may be in the long-term.

## Figures and Tables

**Table 1:** Antioxidant effects on gene expression regulation and their preventive or potential therapeutic value in diseases induced by ionizing radiation, especially when combined with ageing.

Active compounds	Main source	Potential health benefit	Effects on gene expression	References
Polyunsaturated fatty acids (arachidonic acid α-Linolenic acid, omega-3)	Fish oil, algae, green vegetables, flaxseed	Decrease of cardiovascular disease risk. Reduction of serum cholesterol and triacylglycerol. Anti-inflammatory, anti-arrhythmic, anti-thrombotic. Induce membrane fluidity.	Affect the expression of several key proteins pertinent to inflammation, lipid metabolism, and energy utilization.	[100,102,150,151]
Phytosterols	Plants, plant oils	Regulation of cardiovascular disease, anticancer, regulation of serum cholesterol.	Decrease in the expression levels of hepatic genes encoding gluconeogenic enzymes, lipogenic enzymes. Regulation of the expression of gastro-intestinal genes.	[93,94]
Polyphenols (flavonols, anthocyanidins, catechins, isoflavonoids, curcumin)	Apples, onion, tea, grapefruit and orange juice, broccoli	Antioxidant, free radical scavenging metal chelating ability. Antiproliferative and anticarcinogenic agents. Anti-inflammatory activity.	Increase in the expression of endothelial NO synthase and endothelin-1. Curcumin inhibits COX-1 and COX-2 enzymes and reduces the activation of nuclear transcription factor NF-κB.	[116,121,154,155]
Organosulfur compounds (Alliin, allicin)	Garlic, onions	Anti-hypertensive, antithrombotic, anticancer, antimutagenic, antidiabetic, antioxidant, antimicrobial.	Block of the activation of nuclear factor-κB. Blocking the activation of ROS-dependent extracellular regulated kinases (ERK1/2), c-Jun *N*-terminal kinases (JNK1/2) and serine/threonine kinase (AKT).	[109,156,157]
Capsaicinoids (Capsaicin)	Cruciferous vegetables, pepper	Chemopreventive activity, modulation of drug metabolizing enzymes, neuroactivity, apoptotic cell death.	Enhance the transcripts of the proto-oncogenes c-*myc* and c-Ha-*ras* and the tumor suppressor gene *p53.* Induce upregulation of the pro-angiogenetic, pro-invasive and pro-metastatic genes. Modulate adipokine gene expression.	[95,96]
Glucosinolates	Cruciferous plants	Reduce the risk of carcinomas of the lung, stomach, colon and rectum.	Induction of glucoronosyl transferase, glutathione S-transferase, quinone reductase. Induction of cytoprotective genes.	[97,98]
Carotenoids (carotenes, xanthophylls)	Tomatoes, spinach, citrus fruits, carrots	Improvement of visual function, protection from photo-induced damage. Reduce heart disease and cancer.	Modulate the expression of inflammation related genes in retinal pigment epithelial cells.	[135,136,161]

## References

[1] Dainiak N. (2011). Recommendations for assessment of consequences and health risks of low-level exposure to ionizing radiation. Health Phys.

[2] Chen J., Einstein A.J., Fazel R., Krumholz H.M., Wang Y., Ross J.S., Ting H.H., Shah N.D., Nasir K., Nallamothu B.K. (2010). Cumulative exposure to ionizing radiation from diagnostic and therapeutic cardiac imaging procedures: A population-based analysis. J. Am. Coll. Cardiol.

[3] Santiso R., Tamayo M., Gosálvez J., Johnston S., Mariño A., Fernández C., Losada C., Fernández J.L. (2012). DNA fragmentation dynamics allows the assessment of cryptic sperm damage in human: Evaluation of exposure to ionizing radiation, hyperthermia, acidic pH and nitric oxide. Mutat. Res.

[4] Land C.E. (2009). Low-dose extrapolation of radiation health risks: Some implications of uncertainty for radiation protection at low doses. Health Phys.

[5] Hayashi T., Morishita Y., Khattree R., Misumi M., Sasaki K., Hayashi I., Yoshida K., Kajimura J., Kyoizumi S., Imai K. (2012). Evaluation of systemic markers of inflammation in atomic-bomb survivors with special reference to radiation and age effects. FASEB J.

[6] Baker J.E., Moulder J.E., Hopewell J.W. (2011). Radiation as a risk factor for cardiovascular disease. Antioxid. Redox Signal.

[7] Yong L.C., Petersen M.R., Sigurdson A.J., Sampson L.A., Ward E.M. (2009). High dietary antioxidant intakes are associated with decreased chromosome translocation frequency in airline pilots. Am. J. Clin. Nutr.

[8] Damasso M., Dachev T., Falzetta G., Giardi M.T., Rea G., Zanini A. (2009). The radiation environment observed by Liulin-Photo and R3D-B3 spectrum-dosimeters inside and outside Foton-M3 spacecraft. Radiat. Meas.

[9] Stein T.P., Leskiw M.J. (2000). Oxidant damage during and after long duration space flight. FASEB J.

[10] Durante M., Cucinotta F.A. (2008). Heavy ion carcinogenesis and human space exploration. Nat. Rev. Cancer.

[11] Schimmerling W. (2010). Accepting space radiation risks. Radiat. Environ. Biophys.

[12] Cucinotta F.A., Hu S., Schwadron N., Kozarev K., Lawrence T.W., Kim M.H. (2010). Space radiation risk limits and Earth-Moon-Mars environmental models. Space Weather.

[13] Committee to Assess Health Risks from Exposure to Low Levels of Ionizing Radiation; Nuclear and Radiation Studies Board, Division on Earth and Life Studies, National Research Council of the National Academies (2006). Health Risks from Exposure to Low Levels of Ionizing Radiation.

[14] United Nations Scientific Committee on the Effects of Atomic Radiation. Sources and Effects of Ionizing Radiation.

[15] Early and late effects of radiation in normal tissues and organs: Threshold doses for tissue reactions and other non-cancer effects of radiation in a radiation protection context. http://www.icrp.org.

[16] Valko M., Leibfritz D., Moncola J., Cronin M.T.D., Mazura M., Telser J. (2007). Free radicals and antioxidants in normal physiological functions and human disease. Int. J. Biochem. Cell Biol.

[17] Baker J.E., Fish B., Su J., Haworth S.T., Strande J.L., Komorowski R.A., Migrino R.Q., Doppalapudi A., Harmann L., Allen Li X. (2009). 10 Gy total body irradiation increases risk of coronary sclerosis, degeneration of heart structure and function in a rat model. Int. J. Radiat. Biol.

[18] Ziech D., Francor R., Pappa A., Panayiotidis M.I. (2011). Reactive Oxygen Species (ROS)-Induced genetic and epigenetic alterations in human carcinogenesis. Mutat. Res.

[19] Muller H.J., Mott-Smith L.M. (1930). Evidence that natural radioactivity is inadequate to explain the frequency of “natural” mutations. Proc. Natl. Acad. Sci.

[20] Kryston T.B., Georgiev A.B., Pissis P., Georgakilas A.G. (2011). Role of oxidative stress and DNA damage in human carcinogenesis. Mutat. Res.

[21] Dickey J.S., Baird B.J., Redon C.E., Sokolov M.V., Sedelnikova O.A., Bonner W.M. (2009). Intercellular communication of cellular stress monitored by gamma-H2AX induction. Carcinogenesis.

[22] Morgan W.F. (2003). Non-targeted and delayed effects of exposure to ionizing radiation: II. Radiation-induced genomic instability and bystander effects *in vivo*, clastogenic factors and transgenerational effects. Radiat. Res.

[23] Lusis A. (2000). Atherosclerosis. Nature.

[24] Hansson G.K. (2005). Inflammation, atherosclerosis, and coronary artery disease. N. Engl. J. Med.

[25] Cobbold C.A., Sherratt J.A., Maxwell S.R. (2002). Lipoprotein oxidation and its significance for atherosclerosis: A mathematical approach. Bull. Math. Biol.

[26] Little M.P., Gola A., Tzoulaki I. (2009). A model of cardiovascular disease giving a plausible mechanism for the effect of fractionated low-dose ionizing radiation exposure. PLoS Comput. Biol.

[27] Hayashi T., Morishita Y., Kubo Y., Kusunoki Y., Hayashi I., Kasagi F., Hakoda M., Kyoizumi S., Nakachi K. (2005). Long-term effects of radiation dose on inflammatory markers in atomic bomb survivors. Am. J. Med.

[28] Preston D.L., Shimizu Y., Pierce D.A., Suyama A., Mabuchi K. (2003). Studies of mortality of atomic bomb survivors. Report 13: Solid cancer and noncancer disease mortality: 1950–1997. Radiat. Res.

[29] Yamada M., Wong F.L., Fujiwara S., Akahoshi M., Suzuki G. (2004). Noncancer disease incidence in atomic bomb survivors, 1958–1998. Radiat. Res.

[30] Lucas J.N., Poggensee M., Straume T. (1992). The persistence of chromosome translocations in a radiation worker accidentally exposed to tritium. Cytogenet. Cell Genet.

[31] Howe G.R., Zablotska L.B., Fix J.J., Egel J., Buchanan J. (2004). Analysis of the mortality experience amongst U.S. nuclear power industry workers after chronic low-dose exposure to ionizing radiation. Radiat. Res.

[32] Hayashi T., Kusunoki Y., Hakoda M., Morishita Y., Kubo Y., Maki M., Kasagi F., Kodama K., Macphee D.G., Kyoizumi S. (2003). Radiation dose-dependent increases in inflammatory response markers in A-bomb survivors. Int. J. Radiat. Biol.

[33] Vano E., Kleiman N.J., Duran A., Rehani M.M., Echeverri D., Cabrera M. (2010). Radiation cataract risk in interventional cardiology personnel. Radiat. Res.

[34] Hsieh W.A., Lin I.-F., Chang W.P., Chen W.L., Hsu Y.H., Chen M.S. (2010). Lens opacities in young individuals long after exposure to protracted low-dose-rate gamma radiation in ^60^Co-contaminated buildings in Taiwan. Radiat. Res.

[35] Vano E., Kleiman N.J., Duran A., Romano-Miller M., Rehani M.M. (2013). Radiation-associated lens opacities in catheterization personnel: Results of a survey and direct assessments. J. Vasc. Interv. Radiol.

[36] Bonisoli-Alquati A., Mousseau T.A., Møller A.P., Capriolia M., Sainoa N. (2010). Increased oxidative stress in barn swallows from the Chernobyl region. Comp. Biochem. Physiol. Mol. Integr. Physiol.

[37] Beresford N.A., Barnett C.L., Brown J.E., Cheng J.-J., Copplestone D., Gaschak S., Hosseini A., Howard B.J., Kamboj S., Nedveckaite T. (2010). Predicting the radiation exposure of terrestrial wildlife in the Chernobyl exclusion zone: An international comparison of approaches. J. Radiol. Prot.

[38] Pungkun V. (July). Chronic Radiation doses to Aquatic Biota. Ph.D. Thesis.

[39] Bonisoli-Alquati A., Møller A.P., Rudolfsen G., Saino N., Caprioli M., Ostermiller S., Mousseau T.A. (2011). The effects of radiation on sperm swimming behavior depend on plasma oxidative status in the barn swallow *(Hirundo rustica)*. Comp. Biochem. Physiol. Mol. Integr. Physiol.

[40] Chesser R.K., Sugg D.W., Lomakin M.D., van den Bussche R.A., DeWoody J.A., Jagoe C.H., Dallas C.E., Whicker F.W., Smith M.H., Gaschak S.P. (2000). Concentrations and dose rate estimates of ^134,137^cesium and ^90^strontium in small mammals at Chornobyl, Ukraine. Environ. Toxicol. Chem.

[41] Geraskin S.A., Fesenko S.V., Alexakhin R.M. (2008). Effects of non-human species irradiation after the Chernobyl NPP accident. Environ. Int.

[42] Hiyama A., Nohara C., Kinjo S., Taira W., Gima S., Tanahara A., Otaki J.M. (2012). The biological impacts of the Fukushima nuclear accident on the pale grass blue butterfly. Nat. Sci. Rep..

[43] Baqail F.P., Gridleyl D.S., Slater J.M., Luo-Owen X., Stodieck L.S., Ferguson V., Chapes S.K., Pecaut M.J. (2009). Effects of spaceflight on innate immune function and antioxidant gene expression. J. Appl. Physiol.

[44] Fortunati A., Tassone P., Damasso M., Migliaccio F. (2010). Neutron irradiation affects the expression of genes involved in the response to auxin, senescence and oxidative stress in Arabidopsis. Plant Signal. Behav.

[45] Le O.N., Rodier F., Fontaine F., Coppe J.P., Campisi J., de Gregori J., Laverdière C., Kokta V., Haddad E., Beauséjour C.M. (2010). Ionizing radiation-induced long-term expression of senescence markers in mice is independent of p53 and immune status. Aging Cell.

[46] Spitz D.R., Azzam E.I., Li J.J., Gius D. (2004). Metabolic oxidation/reduction reactions and cellular responses to ionizing radiation: A unifying concept in stress response biology. Cancer Metastasis Rev.

[47] Sawant S.G., Randers-Pehrson G., Geard C.R., Brenner D.J., Hall E.J. (2001). The bystander effect in radiation oncogenesis: I. Transformation in C3H 10T1/2 cells *in vitro* can be initiated in the unirradiated neighbors of irradiated cells. Radiat. Res.

[48] Mitchell S.A., Randers-Pehrson G., Brenner D.J., Hall E.J. (2004). The bystander response in C3H 10T1/2 cells: The influence of cell-to-cell contact. Radiat. Res.

[49] Buonanno M., de Toledo S.M., Pain D., Azzam E.I. (2011). Long-term consequences of radiation-induced bystander effects depend on radiation quality and dose and correlate with oxidative stress. Radiat. Res.

[50] Patel R.P., Cornwell T., Darley-Usmar V.M., Cadenas E., Packer L. (1999). The Biochemistry of Nitric Oxide and Peroxynitrite: Implications for Mitochondrial Function. Understanding the Process of Aging: The Roles of Mitochondria, Free Radicals, and Antioxidants.

[51] Zaka R., Vandecasteele C.M., Misset M.T. (2002). Effects of low chronic doses of ionizing radiation on antioxidant enzymes and G6PDH activities in *Stipa capillata (Poaceae)*. J. Exp. Bot.

[52] Esnault M.A.L., Chenal C. (2010). Ionizing radiation: Advances in plant response. Environ. Exp. Bot.

[53] Vandenhove H., Vanhoudt N., Cuypers A., van Hees M., Wannijn J., Horemans N. (2010). Life-cycle chronic gamma exposure of *Arabidopsis thaliana* induces growth effects but no discernable effects on oxidative stress pathways. Plant Physiol. Biochem.

[54] Smith J., Willey N., Hancock J. (2012). Low dose ionising radiation produces too few ROS to directly affect antioxidant concentrations in cells. Biol. Lett.

[55] Finkel T. (2011). Signal transduction by reactive oxygen species. J. Cell Biol.

[56] Prasad K.N., Cole W.C., Kumar B., Prasad K. (2002). Pros and cons of antioxidant use during radiation therapy. Cancer Treat. Rev.

[57] Capizzi R.L., Oster W. (2000). Chemoprotective and radioprotective effects of amifostine: An update of clinical trials. Int. J. Hematol.

[58] Sinclair W.K. (1968). Cysteamine: Differential X-ray protective effect on Chinese hamster cells during the cell cycle. Science.

[59] Anne P.R. (2002). Phase II trial of subcutaneous amifostine in patients undergoing radiation therapy for head and neck cancer. Semin. Oncol.

[60] Sminia P., van der Kracht A.H., Frederiks W.M., Jansen W. (1996). Hyperthermia, radiation carcinogenesis and the protective potential of vitamin A and *N*-acetylcysteine. J. Cancer Res. Clin. Oncol.

[61] Cekan E., Tribukait B., Vokal-Borek D.H. (1985). Protective effect of selenium against radiation induced malformations in mice. Acta Radiol. Oncol.

[62] Kennedy A.R., Krinsky N.I. (1994). Effects of retinoids, b-carotene and canthaxanthene on U.V. and X-ray-induced transformation of C3H10T 1/2 cells *in vitro*. Nutr. Cancer.

[63] Konopacka M., Widel M., Rzeszowska-Wolny J. (1998). Modifying effect of vitamins C, E and beta-carotene against gamma-ray induced DNA damage in mouse cells. Mutat. Res.

[64] Wambi C.O., Sanzari J.K., Sayers C.M., Nuth M., Zhou Z., Davis J., Finnberg N., Lewis-Wambi J.S., Ware J.H., El-Deiry W.S. (2009). Protective effects of dietary antioxidants on proton total-body irradiation-mediated hematopoietic cell and animal survival. Radiat. Res.

[65] Mutlu-Turkoglu U., Erbil Y., Oztezcan S., Olgac V., Toker G., Uysal M. (2000). The effect of selenium and/or vitamin E treatments on radiation-induced intestinal injury in rats. Life Sci.

[66] Ushakova T., Melkonyan H., Nickonova L., Afanasyev V., Gaziev A., Murdrik N., Bradburv R., Goqvadze V. (1999). Modification of gene expression by dietary antioxidants in radiation-induced apoptosis of mice splenocytes. Free Radic. Biol. Med.

[67] Gaziev A., Podlutsky A., Panfilov B., Bradbury R. (1995). Dietary supplements of antioxidants reduce hprt mutant frequency in splenocytes of aging mice. Mutat. Res.

[68] Harapanhalli R.S., Yaghmai V., Giuliani D., Howell R.W., Rao D.V. (1996). Antioxidant effects of vitamin C in mice following X-irradiation. Res. Commun. Mol. Pathol. Pharmacol.

[69] Narra V.R., Harapanhalli R.S., Howell R.W., Sastry K.S., Rao D.V. (1994). Vitamins as radioprotectors *in vivo*. Protection by vitamin C against internal radionuclides in mouse testes: Implications to the mechanism of damage caused by the Auger effect. Radiat. Res.

[70] El-Habit O.H., Saada H.N., Azab K.S., Abdel-Rahman M., El-Malah D.F. (2000). The modifying effect of beta-carotene on gamma radiation-induced elevation of oxidative reactions and genotoxicity in male rats. Mutat. Res.

[71] Boerma M., Roberto K.A., Hauer-Jensen M. (2008). Prevention and treatment of functional and structural radiation injury in the rat heart by pentoxifylline and alpha-tocopherol. Int. J. Radiat. Oncol. Biol. Phys.

[72] Umegaki K., Uramoto H., Suzuki J., Esashi T. (1997). Feeding mice palm carotene prevents DNA damage in bone marrow and reduction of peripheral leukocyte counts, and enhances survival following X-ray irradiation. Carcinogenesis.

[73] Kennedy A.R., Guan J., Ware J.H. (2007). Countermeasures against space radiation induced oxidative stress in mice. Radiat. Environ. Biophys.

[74] Stewart J., Ko Y.H., Kennedy A.R. (2007). Protective effects of L-selenomethionine on space radiation induced changes in gene expression. Radiat. Environ. Biophys.

[75] Sieber F., Muir S.A., Cohen E.P., North P.E., Fish B.L., Irving A.A., Mäder M., Moulder J.E. (2009). High-dose selenium for the mitigation of radiation injury: A pilot study in a rat model. Radiat. Res.

[76] Ben-Amotz A., Yatziv S., Sela M., Greenberg S., Rachmilevich B., Shwarzman M. (1998). Effect of natural beta-carotene supplementation in children exposed to radiation from the Chernobyl accident. Radiat. Environ. Biophys.

[77] Korkina L.G., Afanas’ef I.B., Diplock A.T. (1993). Antioxidant therapy in children affected by irradiation from the Chernobyl nuclear accident. Biochem. Soc. Trans.

[78] Mills E.E. (1988). The modifying effect of beta-carotene on radiation and chemotherapy induced oral mucositis. Br. J. Cancer.

[79] Singh V.K., Brown D.S., Kao T.C. (2009). Tocopherol succinate: A promising radiation countermeasure. Int. Immunopharmacol.

[80] Nakayama A., Alladin K.P., Igbokwe O., White J. (2011). Generating evidence-based guidelines on the concurrent use of dietary antioxidants and chemotherapy or radiotherapy. Cancer Invest.

[81] Davis J.G., Wan X.S., Ware J.H., Kennedy A.R. (2010). Dietary supplements reduce the cataractogenic potential of proton and HZE-particle radiation in mice. Radiat. Res.

[82] Blumenthal R.D., Lew W., Reising A., Soyne D., Osorio L., Ying Z., Goldenberg D.M. (2000). Anti-oxidant vitamins reduce normal tissue toxicity induced by radio-immunotherapy. Int. J. Cancer.

[83] Weiss J.F., Landauer M.R. (2003). Protection against ionizing radiation by antioxidant nutrients and phytochemicals. Toxicology.

[84] Prasad K.N. (2005). Rationale for using multiple antioxidants in protecting humans against low doses of ionizing radiation. Br. J. Radiol.

[85] Devi P.U., Agrawala P.K. (2011). Normal tissue protectors against radiation injury. Def. Sci. J.

[86] Jha M.N., Bedford J.S., Jha S., Prasad K.N. (2011). Caffeine treatment enhances low dose gamma-irradiation-induced chromatid-type aberrations in human leukaemia cells, but not in human normal fibroblast cells in culture. Int. J. Low Radiat.

[87] Krzyzanowska J., Czubacka A., Oleszek W. (2010). Dietary phytochemicals and human. Adv. Exp. Med. Biol.

[88] Rea G., Antonacci A., Lambreva M., Pastorelli S., Ferrari S., Fischer D., Johanningmeier U., Oleszek W., Doroszewska T., Rizzo A.M. (2011). Integrated plant biotechnologies applied to safer and healthier food production: The Nutra-Snack manufacturing chain. Trends Food Sci. Technol.

[89] Fan X.T. (2005). Antioxidant capacity of fresh-cut vegetables exposed to ionizing radiation. J. Sci. Food Agric.

[90] Manda K., Reiter R.J. (2010). Melatonin maintains adult hippocampal neurogenesis and cognitive functions after irradiation. Prog. Neurobiol.

[91] Jagetia G.C. (2007). Radioprotective potential of plants and herbs against the effects of ionizing radiation. J. Clin. Biochem. Nutr.

[92] Arora R., Gupta D., Chawla R., Sagar R., Sharma A., Kumar R., Prasad J., Singh S., Samanta N., Sharma R.K. (2005). Radioprotection by plant products: Present status and future prospects. Phytother. Res.

[93] Misawa E., Tanaka M., Nomaguchi K., Nabeshima K., Yamada M., Toida T., Iwatsuki K. (2012). Oral ingestion of *Aloe vera* phytosterols alters hepatic gene expression profiles and ameliorates obesity-associated metabolic disorders in zucker diabetic fatty rats. J. Agric. Food Chem.

[94] Chen Q., Gruber H., Pakenham C., Ratnayake W.M., Scoggan K.A. (2009). Dietary phytosterols and phytostanols alter the expression of sterol-regulatory genes in SHRSP and WKY inbred rats. Ann. Nutr. Metab.

[95] Caprodossi S., Amantini C., Nabissi M., Morelli M.B., Farfariello V., Santoni M., Gismondi A., Santoni G. (2011). Capsaicin promotes a more aggressive gene expression phenotype and invasiveness in null-TRPV1 urothelial cancer cells. Carcinogenesis.

[96] Kang J.H., Kim C.S., Han I.S., Kawada T., Yu R. (2007). Capsaincin, a spicy component of hot peppers, modulates adipokine gene expression and protein release from obese-mouse adipose tissues and isolated adipocytes, and supresses the inflammatory responses of adipose tissue macrophages. FEBS Lett.

[97] Das S., Srinibas-Tyagi A.K., Kaur H. (2000). Cancer modulation by glucosinolates. Curr. Sci.

[98] Hayes J.D., Kelleher O., Eggelston M. (2008). The cancer chemoprotective actions of phytochemicals derived from glucosinolates. Eur. J. Nutr.

[99] Yokoyama M., Origasa H., Matsuzaki M., Matsuzawa Y., Saito Y., Ishikawa Y., Oikawa S., Sasaki J., Hishida H., Itakura H. (2007). Japan EPA lipid intervention study (JELIS) Investigators. Effects of eicosapentaenoic acid on major coronary events in hypercholesterolaemic patients (JELIS): A randomized open-label, blinded endpoint analysis. Lancet.

[100] Dangour A.D., Allen E., Elbourne D., Fasey N., Fletcher A.E., Hardy P., Holder G.E., Knight R., Letley L., Richards M. (2010). Effect of 2-y n−3 long-chain polyunsaturated fatty acid supplementation on cognitive function in older people: A randomized, double-blind, controlled trial. Am. J. Clin. Nutr.

[101] Gissi H.F., Tavazzi L., Maggioni A.P., Marchioli R., Barlera S., Franzosi M.G., Latini R., Lucci D., Nicolosi G.L., Porcu M. (2008). Effect of n-3 polyunsaturated fatty acids in patients with chronic heart failure (the GISSI-HF trial): A randomised, double-blind, placebo-controlled trial. Lancet.

[102] Fontani G., Corradeschi F., Felici A., Alfatti F., Bugarini R., Fiaschi A.I., Cerretani D., Montorfano G., Rizzo A.M., Berra B. (2005). Blood profiles, body fat and mood state in healthy subjects on different diets supplemented with Omega-3 polyunsaturated fatty acids. Eur. J. Clin. Invest.

[103] McEvoy C., Young I.S., Woodside J.V. (2012). Fish, n-3 polyunsaturated fatty acids, and cardiovascular disease. Nutr. Health.

[104] Lavie C.J., Milani R.V., Mehra M.R., Ventura H.O. (2009). *Omega*-3 polyunsaturated fatty acids and *cardiovascular* diseases. J. Am. Coll. Cardiol.

[105] Chen J., Jiang Y., Liang Y., Tian X., Peng C., Ma K.Y., Liu J., Huang Y., Chen Z.Y. (2012). DPA n-3, DPA n-6 and DHA improve lipoprotein profiles and aortic function in hamsters fed a high cholesterol diet. Atherosclerosis.

[106] Mazza M., Pomponi M., Janiri L., Bria P., Mazza S. (2007). Omega-3 fatty acids and antioxidants in neurological and psychiatric diseases: An overview. Prog. Neuro-psychopharmacol. Biol. Psychiatry.

[107] Chiu C.-C., Su K.-P., Cheng T.-C., Liu H.C., Chang C.J., Dewey M.E., Stewart R., Huang S.Y. (2008). The effects of omega-3 fatty acids monotherapy in Alzheimer’s disease and mild cognitive impairment: A preliminary randomized double-blind placebo-controlled study. Prog. Neuropsychopharmacol. Biol. Psychiatry.

[108] Beltz B.S., Tlusty M.F., Benton J.L., Sandeman D.C. (2007). Omega-3 fatty acids upregulate adult neurogenesis. Neurosci. Lett.

[109] Rogers P.J., Appleton K.M., Kessler D., Peters T.J., Gunnell D., Hayward R.C., Heatherley S.V., Christian L.M., McNaughton S.A., Ness A.R. (2008). No effect of n-3 *l*ong-chain polyunsaturated fatty acid (EPA and DHA) supplementation on depressed mood and cognitive function: A randomized controlled trial. Br. J. Nutr.

[110] Jones P.J.H., AbuMweis S.S. (2009). Phytosterols as functional food ingredients: Linkages to cardiovascular disease and cancer. Curr. Opin. Clin. Nutr. Metab. Care.

[111] Liang G., Qiao X., Bi Y., Zou B., Zheng Z. (2012). Studies on purification of allicin by molecular distillation. J. Sci. Food Agric.

[112] Liu C., Cao F., Tang Q.-Z., Yan L., Dong Y.G., Zhu L.H., Wang L., Bian Z.Y., Li H. (2010). Allicin protects against cardiac hypertrophy and fibrosis via attenuating reactive oxygen species-dependent signaling pathways. J. Nutr. Biochem.

[113] Touloupakis E., Ghanotakis D.F. (2010). Nutraceutical use of garlic sulfur-containing compounds. Adv. Exp. Med. Biol.

[114] Li X.H., Li C.Y., Lu J.M., Tian R.B., Wei J. (2012). Allicin ameliorates cognitive deficits ageing-induced learning and memory deficits through enhancing of Nrf2 antioxidant signaling pathways. Neurosci. Lett.

[115] Wu C.C., Chung J.G., Tsai S.J., Yang J.H., Sheen L.Y. (2004). Differential effects of allyl sulfides from garlic essential oil on cell cycle regulation in human liver tumor cells. Food Chem. Toxicol.

[116] Zhang R., Humphreys I., Sahu R.P., Shi Y., Srivastava S.K. (2008). *In vitro* and *in vivo* induction of apoptosis by capsaicin in pancreatic cancer cells is mediated through ROS generation and mitochondrial death pathway. Apoptosis.

[117] Traka M., Mithen R. (2009). Glucosinolates, isothiocyanates and human health. Phytochem. Rev.

[118] Arts I.C.W., Hollman P.C.H. (2005). Polyphenols and disease risk in epidemiologic studies. Am. J. Clin. Nutr.

[119] Loffredo L., Violi F. (2012). Polyphenolic Antioxidants and Health. Chocolate and Health.

[120] Cordova A.C., Sumpio B.E. (2009). Polyphenols are medicine: Is it time to prescribe red wine for our patients?. Int. J. Angiol.

[121] Kuriyama S. (2008). The relation between green tea consumption and cardiovascular disease as evidenced by epidemiological studies. J. Nutr.

[122] Lin J.-K., Weng M.-S., Grotewold E. (2006). Flavonoids as Nutraceuticals. The Science of Flavonoids.

[123] Shen W.H., Balajee A.S., Wang J., Wu H., Eng C., Pandolfi P.P., Yin Y. (2007). Essential role for nuclear PTEN in maintaining chromosomal integrity. Cell.

[124] Nguyen T., Sherratt P.J., Pickett C.B. (2003). Regulatory mechanisms controlling gene expression mediated by the antioxidant response element. Annu. Rev. Pharmacol. Toxicol.

[125] Cho J.W., Park K., Kweon G.R., Jang B.C., Baek W.K., Suh M.H., Kim C.W., Lee K.S., Suh S.I. (2005). Curcumin inhibits the expression of COX-2 in UVB-irradiated human keratinocytes (HaCaT) by inhibiting activation of AP-1: p38 MAP kinase and JNK as potential upstream targets. Exp. Mol. Med.

[126] Epstein J., Docena G., MacDonald T.T., Sanderson I.R. (2010). Curcumin suppresses p38 mitogenactivated protein kinase activation, reduces IL1β and matrix metalloproteinase and enhances IL10 in the mucosa of children and adults with inflammatory bowel disease. Br. J. Nutr.

[127] Scapagnini G., Caruso C., Calabrese V. (2010). Therapeutic potential of dietary polyphenols against brain ageing and neurodegenerative disorders. Adv. Exp. Med. Biol.

[128] Srinivasan M., Rajendra-Prasad N., Menon V.P. (2006). Protective effect of curcumin on gamma-radiation induced DNA damage and lipid peroxidation in cultured human lymphocytes. Mutat. Res.

[129] Carsten R.E., Bachand A.M., Bailey S.M., Ullrich R.L. (2008). Resveratrol reduces radiation-induced chromosome aberration frequencies in mouse bone marrow cells. Radiat. Res.

[130] Berendschot T., Plat J., de Jong A., Mensink R.P. (2009). Longterm plant stanol and sterol ester enriched functional food consumption, serum lutein/zeaxanthin concentration and macular pigment optical density. Br. J. Nutr.

[131] Graydon R., Hogg R.E., Chakravarthy U., Young I.S., Woodside J.V. (2012). The effect of lutein- and zeaxanthin-rich foods v. supplements on macular pigment level and serological markers of endothelial activation, inflammation and oxidation: Pilot studies in healthy volunteers. Br. J. Nutr.

[132] Barker F.M., Snodderly D.M., Johnson E.J., Schalch W., Koepcke W., Gerss J., Neuringer M. (2011). Nutritional manipulation of primate retinas, v: Effects of lutein, zeaxanthin, and n–3 fatty acids on retinal sensitivity to blue-light-induced damage. Invest. Ophthalmol. Vis. Sci.

[133] Smith S.M., Zwart S.R., Block G., Rice B.L., Davis-Street J.E. (2005). The nutritional status of astronauts is altered after long-term spaceflight aboard the International Space Station. J. Nutr.

[134] Casolino M., Bidoli V., Morselli A., Narici L., de Pascale M.P., Picozza P., Reali E., Sparvoli R., Mazzenga G., Ricci M. (2003). Space travel: Dual origins of light flashes seen in space. Nature.

[135] Narici L., de Martino A., Brunetti V., Rinaldi A., Sannita W.G., Paci M. (2009). Radicals excess in the retina: A model for light flashes in space. Radiat. Meas.

[136] Narici L., Paci M., Brunetti V., Rinaldi A., Sannita W.G., de Martino A. (2012). Bovine rod rhodopsin. 1. Bleaching by luminescence *in vitro* by recombination of radicals from polyunsaturated fatty acids. Free Radic. Biol. Med.

[137] Roberts R.L., Green J., Lewis B. (2009). Lutein and zeaxanthin in eye and skin health. Clin. Dermatol.

[138] Cho E., Hankinson S.E., Rosner B., Willett W.C., Colditz G.A. (2008). Prospective study of lutein/zeaxanthin intake and risk of age-related macular degeneration. Am. J. Clin. Nutr.

[139] Terman A., Brunk U.T. (1998). Lipofuscin: Mechanisms of formation and increase with age. APMIS.

[140] Ma L., Lin X.M. (2010). Effects of lutein and zeaxanthin on aspects of eye health. J. Sci. Food Agric.

[141] Sasaki M., Yuki K., Kurihara T., Miyake S., Noda K., Kobayashi S., Ishida S., Tsubota K., Ozawa Y. (2012). Biological role of lutein in the light-induced retinal degeneration. J. Nutr. Biochem.

[142] Kaya S., Weigert G., Pemp B., Sacu S., Werkmeister R.M., Dragostinoff N., Garhöfer G., Schmidt-Erfurth U., Schmetterer L. (2012). Comparison of macular pigment in patients with age-related macular degeneration and healthy control subjects—A study using spectral fundus reflectance. Acta Ophthalmol.

[143] Rea G., Esposito D., Damasso M., Serafini A., Margonelli A., Faraloni C., Torzillo G., Zanini A., Bertalan I., Johanningmeier U. (2008). Ionizing radiation impacts photochemical quantum yield and oxygen evolution activity of Photosystem II. Int. J. Radiat. Biol.

[144] Rea G., Lambreva M., Polticelli F., Bertalan I., Antonacci A., Pastorelli S., Damasso M., Johanningmeier U., Giardi M.T. (2011). Directed evolution and in silico analysis of reaction centre proteins reveal molecular signatures of photosynthesis adaptation to radiation pressure. PLoS One.

[145] Kreimer G. (2009). The green algal eyespot apparatus: A primordial visual system and more?. Curr. Genet.

[146] Giardi M.T. IC-CNR Area della ricerca di Roma, Monterotondo scalo 00015, Italy.

[147] Socha K.K., Souza G.A., Ebaid G.M.X., Seiva F.R., Cataneo A.C., Novelli E.L. (2009). Resveratrol toxicity: Effects on risk factors for atherosclerosis and hepatic oxidative stress in standard and high-fat diets. Food Chem. Toxicol.

[148] Suh K.S., Chon S., Oh S., Kim S.W., Kim J.-W., Kim Y.S., Woo J.-T. (2010). Prooxidative effects of green tea polyphenol (−)-epigallocatethin-3-gallate on the HIT-T15 pancreatic beta cell line. Cell Biol. Toxicol.

[149] Jahangir M., Abdel-Farid I.B., Kim H.K., Choia Y.H., Verpoortea R. (2009). Healthy and unhealthy plants: The effect of stress on the metabolism of *Brassicaceae*. Environ. Exp. Bot.

[150] Aherne S.A., O’Brien N.M. (2002). Dietary flavonols: Chemistry, food content, and metabolism. Nutrition.

[151] Satia J.A., Littman A., Slatore C.G., Galanko J.A., White E. (2009). Long-term use of β-carotene, retinol, lycopene, and lutein supplements and lung cancer risk: Results from the vitamins and lifestyle (VITAL) study. Am. J. Epidemiol.

[152] Mikkelsen C.S., Mikkelsen D.B., Lindegaard H.M. (2009). Carotinaemia in patient with excessive beta-carotene food-intake and dysregulated diabetes mellitus. Ugeskr. Laeger.

[153] Weigert G., Kaya S., Pemp B., Sacu S., Lasta M., Werkmeister R.M., Dragostinoff N., Simader C., Garhöfer G., Schmidt-Erfurth U. (2011). Effects of lutein supplementation on macular pigment optical density and visual acuity in patients with age-related macular degeneration. Invest. Ophthalmol. Vis. Sci.

[154] Marangoni F., Poli A. (2010). Phytosterols and cardiovascular health. Pharmacol. Res.

[155] Lee J.Y., Hwang D.H. (2002). Docosahexaenoic acid suppresses the activity of peroxisome proliferator-activated receptors in a colon tumor cell line. Biochem. Biophys. Res. Commun.

[156] Kew S., Wells S., Thies F., McNeill G.P., Quinlan P.T., Clark G.T., Dombrowsky H., Postle A.D., Calder P.C. (2003). The effect of eicosapentaenoic acid on rat lymphocyte proliferation depends upon its position in dietary triacylglycerols. J. Nutr.

[157] Scalbert A., Manach C., Morand C., Rémésy C., Jiménez L. (2005). Dietary polyphenols and the prevention of diseases. Crit. Rev. Food Sci. Nutr.

[158] Nicholson S.K., Tucker G.A., Brameld J.M. (2008). Effects of dietary polyphenols on gene expression in human vascular endothelial cells. Proc. Nutr. Soc.

[159] Jakubowski H. (2003). On the health benefits of *Allium* sp. Nutrition.

[160] Corzo-Martinez M., Corzo N., Villamiel M. (2007). Biological properties of onions and garlic. Trends Food Sci. Technol.

[161] Bian Q., Gao S., Zhou J., Qin J., Taylor A., Johnson E.J., Tang G., Sparrow J.R., Gierhart D., Shang F. (2012). Lutein and zeaxanthin supplementation reduces photooxidative damage and modulates the expression of inflammation-related genes in retinal pigment epithelial cells. Free Radic. Biol. Med.

[162] Prasad K.N., Cole W.C., Haase G.M. (2004). Radiation protection in humans: Extending the concept of as low as reasonably achievable (ALARA) from dose to biological damage. Br. J. Radiol.

